# Effects of leukocyte elastase in semen on sperm quality

**DOI:** 10.1097/MD.0000000000031111

**Published:** 2022-10-14

**Authors:** Qingtai Wang, Chengwen Que, Gangxin Chen

**Affiliations:** a The Second Affiliated Hospital of Fujian University of Traditional Chinese Medicine, Medical Laboratory, Fuzhou, China; b Fujian Provincial Maternity and Children Hospital, Affiliated to Fujian Medical University Medical Laboratory, Fu Zhou, China; c Fujian Provincial Maternity and Children Hospital, Affiliated to Fujian Medical University, Assisted Reproduction Laboratory, Fu Zhou, China.

**Keywords:** leukocyte elastase, sperm DFI, sperm kinetics, sperm trajectory sperm morphology

## Abstract

The study analyzed the effect of leukocyte elastase (LE) in 460 semen on sperm quality, and explore the reference interval of normal level of LE in semen. The differences of LE levels between normal semen and few, weak and abnormal semen were analyzed. Referring to domestic standards, the samples were divided into normal group (LE ≤ 250 ng/mL), occult infection (250 < LE ≤ 1000 ng/mL), and infection group (LE > 1000 ng/mL), and the differences in semen quality among the groups were compared. According to European standards, the samples were divided into normal group (≤600 ng/mL) and abnormal group (>600 ng/mL), and the differences in semen quality between the 2 groups were compared. The correlation between LE levels in semen and semen quality were analyzed. The positive rates of LE in the normal semen group and abnormal semen groups were 30.7% versus 34.7%, and there was no significant difference between the two groups (*P* > .05). When the semen divided into 3 groups, there was no significant difference between the physicochemical parameters, kinetic parameters, movement trajectory parameters, morphological parameters, and sperm DNA fragmentation index (DFI) (*P* > .05). There were significant differences in sperm morphology and sperm DFI between the two groups at 600 ng/mL (*P* < .05). Spearman correlation analysis showed that there was no significant difference between the level of LE in semen and physicochemical parameters, sperm kinetic parameters, sperm movement trajectory parameters, sperm morphological parameters, and sperm DFI (*P* > .05). It is appropriate to use 600 ng/mL as the threshold for the concentration of LE in semen; the correlation between the level of LE and sperm quality is not significant.

## 1. Introduction

Reproductive tract infection is an important cause of male infertility, and anti-inflammatory treatment is an important means to improve male fertility.^[1,2]^ The increase of leukocytes in semen may be a sign of reproductive system infection. When the concentration of leukocytes is more than 1 × 10^6^/mL, sperm quality will decrease.^[3]^ However, we often have the phenomenon that leukocytes in semen do not increase after genital tract infection during clinical diagnosis. At this time, we need to detect the level of leukocyte elastase (LE) in seminal plasma to confirm whether the infection is infected. LE in semen is an important marker of male genital tract infection, and its content will increase significantly when genital tract infection occurs.^[4]^ The aim of this study was to investigate the effect of LE on sperm quality and reference intervals for LE levels in semen.

## 2. Materials and Methods

### 2.1. Information

A total of 460 semen samples from patients who were treated in Fujian Maternal and Child Health Hospital in 2021 were selected, and their LE levels and sperm quality-related parameters (including semen physicochemical parameters, kinetic parameters, motion trajectory parameters, morphological parameters, and DNA fragmentation index (DFI)) were analyzed.

The study was approved by the ethics review board of the Assisted Reproduction Laboratory Fujian Provincial Maternity and Children Hospital, Affiliated to Fujian Medical University.

### 2.2. Method

Patients with semen collection should abstain from sex for 2 to 7 days before semen collection. Semen was collected by masturbation, and the volume of semen was measured by weighing method.^[3]^ The samples were placed in a 35 °C incubator to liquefy for 30 to 60 minute.

Sperm dynamics and movement trajectory analysis referring to the guidance method in the semen examination manual,^[3]^ the computer-aided analysis (CASA) method (Shanghai Beiang Pharmaceutical Technology Co., Ltd.) was used to detect the concentration of sperm, activity, and movement trajectory.

Sperm morphological analysis referring to the Semen Examination Manual,^[3]^ Diff-Quick staining kit (Anke Biotechnology Co., Ltd.) was used for cytological staining. Observe more than 200 spermatozoa under the microscope, and evaluate the normal morphology rate of spermatozoa, the deformity rate of head, neck and middle segment, and main segment.^[5]^

Sperm DNA integrity analysis sperm chromatin diffusion (SCD) method was used for staining (Anke Biotechnology Co., Ltd.). Observe more than 500 sperms under the microscope. Assess the integrity of sperm DNA. DFI (DFI = number of sperm without halo or small halo/total number of counted sperm × 100%).

Detection of LE concentration in seminal plasma after centrifugation of the remaining semen, enzyme linked immunosorbent assay (ELISA) kit (Shenzhen Boruid Biotechnology Co., Ltd.) was used to label LE antibodies, and the LE antibody was labeled in a microplate reader with a wavelength of 450 nm (Shenzhen Boruid Biotechnology Co., Ltd. BRED-2900) to detect the concentration of LE in seminal plasma.

Grouping semen samples were divided into normal group (≤250 ng/mL), occult infection group (250–1000 ng/mL) and infection group (>1000 ng/mL) according to LE concentration; the differences in sperm quality between groups were compared. The samples were divided into normal group (≤600 ng/mL) and abnormal group (>600 ng/mL) according to European standards; the differences in sperm quality between the 2 groups were compared.

### 2.3. Statistical analysis

Data analysis was performed using SPSS 26.0 statistical software. The measurement data that conform to the normal distribution is expressed as mean ± standard deviation (s.d.), and the independent samples t test is used for comparison between groups; the one-way analysis of variance analysis (ANOVA-LSD) was used to determine the significant difference of means among the groups; the measurement data that does not conform to the normal distribution is expressed as M (P25, P75), and the Kruskal–Wallis nonparametric test is used for the comparison between groups; counts the data were expressed as rate (%), and the continuous-corrected chi-square test was used for comparison between groups. Correlation analysis was performed using Spearman test, and the correlation coefficient was represented by r. *P* < .05 indicates that the results are statistically significant.

## 3. Results

### 3.1. Overall data

**Figure 1.**
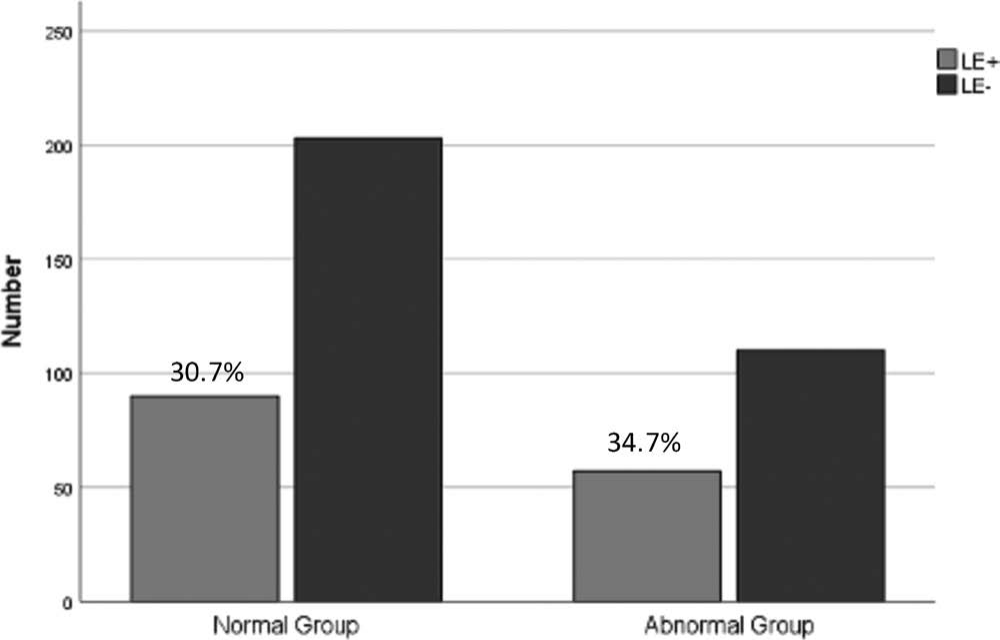
Comparison of i.e. (>600 ng/mL) between the different semen. The positive rate of LE in normal group is 30.7%, and in abnormal group is 34.7% (*P* < .05). LE = leukocyte elastase.

The average age of the 460 patients was 33.5 ± 5.5 years old, the abstinence time was 4.3 ± 1.3 days, the semen volume was 3.1 ± 1.4 mL, the sperm concentration was 79.37 ± 53.45 × 10^6^/mL, and the total sperm motility was 50.1% ± 24.8%, normal sperm morphology rate was 7.4% ± 4.1%, sperm DFI was 11.4% ± 7.7%; all the results were within the normal reference range recommended by WHO. Among them, there were 293 cases of semen with completely normal quality; 167 cases of small, weak and deformed semen. The positive rate of LE between the two groups was 30.7% versus 34.7%, and the difference was not statistically significant (*P* < .05) see Figure 1.

### 3.2. The semen quality comparison between the 2 groups with LE600 ng/mL as the boundary

There was no statistical difference between the 2 groups in parameters such as semen volume, sperm dynamics, and sperm movement trajectory (*P* > .05). The normal sperm morphology rate in the normal group was higher than that in the abnormal group, and the sperm deformity rate in each part was lower than that in the abnormal group; the sperm DFI in the normal group was lower than that in the abnormal group, and the differences were statistically significant (*P* < .05) (see Table 1).

**Table 1:** Comparison of sperm quality in 2 different LE (n = 460).

Sperm parameter	LE (ng/mL)			
	Normal group (<600) n = 312	Inflammation group (≥600) n = 148	t	*P* value
Age (yr)	33.3 ± 5.4	33.9 ± 5.5	–0.965	.335
Abstinence (day)	4.3 ± 1.3	4.3 ± 1.2	0.470	.639
Volume (mL)	3.2 ± 1.4	3.0 ± 1.4	1.410	.159
Concentration (10^6^/mL) M (P25, P75)	66.9 (40.4, 109.5)	67.6 (38.1, 106.8)	0.561	.575
Sperm PR (%) M (P25, P75)	43.6 (24.4, 58.8)	37.9 (22.0, 56.1)	1.255	.210
Total motility (%) M (P25, P75)	53.2 (30.2, 72.6)	45.6 (26.0, 73.4)	1.388	.166
VCL (μm/s)	65.0 ± 17.7	63.2 ± 16.7	1.064	.288
VSL (μm/s)	30.6 ± 9.3	29.8 ± 8.7	0.836	.403
VAP (μm/s)	35.9 ± 10.3	34.7 ± 9.7	1.150	.251
ALH (μm)	2.0 ± 0.7	1.9 ± 0.7	1.134	.257
LIN (CSL/VCL %)	47.9 ± 10.5	47.1 ± 11.7	0.705	.481
WOB (VAP/VCL %)	55.6 ± 9.5	54.8 ± 10.5	0.790	.430
STR (VSL/VAP %)	80.9 ± 10.5	80.0 ± 12.6	0.765	.444
BCF (HZ)	4.8 ± 0.7	4.7 ± 0.7	0.599	.549
Normal form rate (%)	7.7 ± 4.2	6.7 ± 3.7	2.527	.012
Head deformity rate (%)	89.6 ± 5.9	90.9 ± 5.3	–2.123	.034
Middle section deformity rate (%)	18.5 ± 7.3	20.0 ± 7.5	–2.033	.043
Primary segment deformity rate (%)	7.4 ± 5.4	8.2 ± 5.6	–1.484	.139
Sperm DFI (%)	10.9 ± 7.2	12.4 ± 8.5	–2.017	.044

DFI = DNA fragmentation index, LE = leukocyte elastase.

There were no differences in physicochemical parameters and sperm kinetic parameters between the 2 groups. However, there were significant differences in sperm morphological parameters and DNA integrity parameters between the 2 groups.

### 3.3. The semen quality comparison of the 3 groups with LE250 ng/mL and 1000 ng/mL as the boundary

The semen volume and sperm VSL of the 3 groups were statistically different (*P* < .05). There were no significant differences in sperm concentration, sperm dynamics, sperm morphology, sperm DFI, and other parameters among the 3 groups (*P* > .05) (see Table 2).

**Table 2:** Comparison of sperm quality in 3 different LE (n = 460).

Sperm parameter	LE (ng/mL)			F	*P*
	Normal group (<250) n = 247	Occult inflammation group (250–1000) n = 107	Inflammation group (>1000) n = 106		
Age (yr)	33.2 ± 5.5	33.7 ± 5.2	34.1 ± 5.7	1.102	.333
Abstinence (day)	4.4 ± 1.3	4.2 ± 1.3	4.4 ± 1.3	0.966	.381
Volume (mL)	3.2 ± 1.5	3.1 ± 1.2	2.9 ± 1.5	4.789	.029
Concentration (10^6^/mL) M (P25, P75)	64.3 (39.2, 104.1)	68.1 (37.0, 119.4)	68.8 (43.7, 109.4)	0.045	.956
Sperm PR (%) M (P25, P75)	41.7 (24.0, 57.4)	43.5(20.8, 59.2)	40.4 (26.0, 57.1)	0.072	.931
Total motility (%) M (P25, P75)	52.1 (29.3, 72.5)	48.6 (28.7, 69.0)	51.9 (28.1, 74.4)	0.376	.687
VCL (μm/s)	64.3 ± 18.3	66.9 ± 16.4	62.1 ± 16.0	2.005	.136
VSL (μm/s)	30.1 ± 9.2	32.1 ± 9.3	29.1 ± 8.5	3.134	.044
VAP (μm/s)	35.3 ± 10.3	37.3 ± 10.1	34.1 ± 9.5	2.824	.060
ALH (μm)	2.0 ± 0.7	2.0 ± 0.6	1.9 ± 0.6	1.092	.337
LIN (CSL/VCL %)	48.0 ± 10.5	47.7 ± 10.6	46.5 ± 12.1	0.784	.457
WOB (VAP/VCL %)	55.6 ± 9.2	55.6 ± 10.1	54.4 ± 11.1	0.659	.518
STR (VSL/VAP %)	81.2 ± 10.3	80.4 ± 10.1	79.6 ± 13.9	0.779	.459
BCF (HZ)	4.7 ± 0.7	4.8 ± 0.6	4.7 ± 0.8	0.929	.396
Normal form rate (%)	7.6 ± 4.1	7.1 ± 4.1	7.2 ± 4.0	0.890	.410
Head deformity rate (%)	89.7 ± 5.9	90.6 ± 5.7	90.3 ± 5.5	1.111	.330
Middle section deformity rate (%)	18.7 ± 7.3	19.3 ± 7.1	19.6 ± 7.9	0.716	.489
Primary segment deformity rate (%)	7.5 ± 5.4	7.7 ± 5.1	8.0 ± 5.9	0.344	.709
Sperm DFI (%)	10.9 ± 6.9	12.0 ± 8.5	11.9 ± 8.5	1.168	.312

DFI = DNA fragmentation index, LE = leukocyte elastase.

After grouping LE with 250 ng/mL and 1000 ng/mL as the boundary, it can be seen that there is no difference in other main parameters except for the volume and part of the movement trajectory of the semen between the 3 groups.

### 3.4. Comparison of the correlation between LE levels in semen and related sperm parameters

There was no significant correlation between LE levels in semen and semen physicochemical parameters, sperm kinetic parameters, sperm morphological parameters, and sperm DFI (*P* > .05); sperm dynamics, sperm morphology, sperm DFI, and other parameters were significantly correlated (*P* < .05) (see Table 3).

**Table 3:** Correlation between LE and sperm quality (n = 460).

Sperm parameter	Value (mean ± s.d.)	LE (ng/mL)		Concentration(10^6^/mL)		Sperm PR (%)		Normal rate (%)		Sperm DFI (%)	
		797.7 ± 1322.6		79.4 ± 53.4		41.5 ± 20.9		7.4 ± 4.1		11.4 ± 7.7	
		r	*P*	r	*P*	r	*P*	r	*P*	r	*P*
Age (yr)	33.5 ± 5.5	0.068	.144	0.051	.273	–0.110	.018	–0.064	.169	0.134	.004
Abstinence (d)	4.3 ± 1.3	–0.008	.858	0.083	.076	–0.003	.942	–0.030	.523	0.099	.034
Volume (mL)	3.1 ± 1.4	–0.066	.157	–0.161	.001	0.018	.707	–0.021	.653	0.002	.973
Sperm concentration(10^6^/mL)	79.4 ± 53.4	0.021	.648	1.000	–	0.518	.000	0.485	.000	–0.300	.000
Sperm PR (%)	41.5 ± 20.9	0.013	.784	0.518	.000	1.000	–	0.648	.000	–0.593	.000
Total sperm motility (%)	50.1 ± 24.8	0.006	.905	0.031	.091	0.991	.000	0.559	.000	–0.551	000
VCL (μm/s)	64.4 ± 17.4	–0.036	.440	0.210	.000	0.425	.000	0.317	.000	–0.327	.000
VSL (μm/s)	30.3 ± 9.1	0.030	.522	0.124	.008	0.460	.000	0.336	.000	–0.385	.000
VAP (μm/s)	35.5 ± 10.1	0.015	.752	0.207	.000	0.494	.000	0.383	.000	–0.386	.000
ALH (μm)	2.0 ± 0.7	–0.059	.207	0.284	.000	0.404	.000	0.341	.000	–0.320	.000
LIN (CSL/VCL %)	47.6 ± 10.9	0.016	.724	–0.171	.000	–0.171	.000	0.049	.297	–0.147	.002
WOB (VAP/VCL %)	55.3 ± 9.8	0.035	.454	–0.033	.483	0.268	.000	0.124	.008	–0.182	.000
STR (VSL/VAP %)	80.6 ± 11.2	–0.008	.871	–0.340	.000	–0.171	.000	–0.064	.169	–0.056	.229
BCF (HZ)	4.7 ± 0.7	0.022	.637	–0.238	.000	–0.016	.739	0.003	.953	–0.063	.181
Normal form rate (%)	7.4 ± 4.1	–0.068	.144	0.485	.000	0.648	.000	1.000	–	–0.628	.000
Head deformity rate (%)	90.0 ± 5.7	0.074	.114	–0.477	.000	–0.589	.000	–0.906	.000	0.608	.000
Middle section deformity rate (%)	19.0 ± 7.4	0.065	.162	–0.370	.000	–0.530	.000	–0.709	.000	0.517	.000
Primary segment deformity rate (%)	7.7 ± 5.5	0.019	.669	–0.341	.000	–0.475	.000	–0.637	.000	0.510	.000
Sperm DFI (%)	11.4 ± 7.7	0.020	.674	–0.300	.000	–0.593	.000	–0.628	.000	1.000	–

DFI = DNA fragmentation index, LE = leukocyte elastase.

Spearman test showed significant correlations between semen concentration, sperm dynamics, sperm morphology, sperm DFI, and other parameters.

## 4. Discussion

LE in semen is a metal ion-dependent proteolytic enzyme, and its content is the highest in neutrophils, and the increase in its secretion marks the inflammatory manifestations of the reproductive tract and its accessory glands.^[5]^ At present, conclusions about the effect of LE in semen on sperm quality are less consistent. Most studies suggest that the increase of LE in semen can seriously affect sperm quality,^[6,7]^ but there are also studies showing that it has a limited effect on sperm quality.^[8,9]^ Some studies suggest that LE can be used as a marker for “occult” infection of the reproductive system, while others suggest that it has no clinical significance for the etiological analysis of male infertility.^[10–12]^ These differences may be due to different methods of detection of LE in different studies, different study populations, and different selection of LE cutoff values.

Our results showed that the positive rate of LE in normal and abnormal semen was 30.7% versus 34.7%, respectively, and there was no statistical difference between the 2 (*P* > .05), which is roughly the same as the conclusion of the current study.^[11,13]^ When studying the relationship between LE level and sperm quality, we refer to the studies in related literature,^[6,14,15]^ and set the semen LE level as 250 ng/mL and 1000 ng/mL as the boundary. There were no significant differences in semen physicochemical parameters, sperm motility and trajectory, sperm morphology, sperm DFI and other parameters among the three groups (*P* > .05). This result is inconsistent with some research results,^[6,15]^ but basically consistent with some research conclusions.^[8,9]^ When we refer to the normal reference range recommended by the European Society of Urology,^[16]^ when the LE level is divided into normal and abnormal groups with 600 ng/mL as the boundary, it can be seen that the sperm morphology and DFI of the normal group are compared with the abnormal group. difference (*P* < .05), which is almost consistent with Reinhardt results.^[17]^ The results indicate that sperm quality is affected when LE levels in semen are higher than 600 ng/mL. In general, LE can damage sperm through 2 pathways: First, it can directly damage sperm by inducing phagocytosis, apoptosis, etc by regulating cytokines and reactive oxygen species (ROS). The increase of LE level is mostly caused by the increase of neutrophils, and the increase of leukocytes leads to the increase of ROS production.^[18]^ Studies have shown that the ROS generated by activated leukocytes is 10 times that of the normal state.^[19]^ A moderate amount of ROS can promote sperm quality (such as acrosome reaction, sperm capacitation), but excessive ROS can lead to peroxidation of cell membranes and organelles containing unsaturated fatty acids, resulting in sperm damage.^[20]^ On the other hand, it can damage the function of the epididymis and accessory gonads through inflammation, thereby indirectly causing male infertility.^[21]^

Spearman analysis showed that the concentration of LE in normal semen had little correlation with sperm quality, which was roughly the same as related reports.^[10–12]^ The reasons for the small correlation between the level of LE in semen and sperm-related quality parameters may be as follows: The increase of LE is mainly caused by the infection of accessory gonads such as the prostate, and the formation of sperm is mainly determined by the internal environment of the testis. The quality parameters of semen do not necessarily reflect the degree of infection in the reproductive system.^[22]^ Only activated leukocytes can lead to the increase of LE, and some leukocytes in the samples of this study were not fully activated. Because individual responses to infection vary widely, some patients are more resistant to LE.^[23]^

There are many detection methods for LE, including radioimmunoassay, chemiluminescence, immunoprecipitation, enzyme-linked immunosorbent assay, and electrophoresis. The ELISA method is widely used because it can be operated in batches and the method is relatively stable. The detection methods of semen LE still need to be standardized, and the diagnostic criteria still need to be unified. Most of the previous studies set the semen LE < 250 ng/mL as a normal level, LE > 1000 ng/mL as a marker of reproductive system infection, and between 250–1000 ng/mL as a marker of occult infection in the reproductive system.^[14,15,24]^ There are also reports that the critical points of genital tract inflammation are set at 230 ng/mL,^[10]^ 290 ng/mL,^[25]^ and 600 ng/mL.^[16]^ This study compared the effect of 250 ng/mL, 1000 ng/mL, and 600 ng/mL as the cutoff point on sperm quality and found that it may be more scientific to use 600 ng as the cutoff point for LE. We also analyzed the correlation between sperm motility, morphology, and DFI. The results of the analysis found that these data are interrelated and consistent with the current related research results,^[26,27]^ which indirectly proves the reliability of our experimental data.

## 6. Conclusions

The results of this study also show that sperm quality does not necessarily deteriorate when an occult infection (250–1000 ng/mL) of the reproductive tract occurs. The result may be due to the different tolerance of LE concentrations in different individuals. It’s also the reason for inconsistent results of previous studies on the correlation between LE and sperm quality. Therefore, the determination of LE occult infection indicators can only be determined by subdividing the population according to the actual situation. Because of the small sample size of the study and the many factors affecting sperm quality, only the univariate analysis of the correlation between LE and sperm quality has some limitations.

## Author contributions

Gangxin Chen designed the research, performed the research, analyzed the data, and wrote the manuscript. Qingtai Wang and Chengwen Que collected the data, searched the literature, finished language editing, project designed. All authors read and approved the final manuscript.

**Conceptualization:** Qingtai Wang, Gangxin Chen.

**Data curation:** Qingtai Wang, Gangxin Chen.

**Formal analysis:** Qingtai Wang.

**Funding acquisition:** Qingtai Wang.

**Project administration:** Chengwen Que.

**Resources:** Chengwen Que.

**Software:** Gangxin Chen.

**Supervision:** Chengwen Que.

**Validation:** Gangxin Chen.

**Visualization:** Chengwen Que.

**Writing – original draft:** Chengwen Que, Gangxin Chen.

**Writing – review & editing:** Gangxin Chen.
